# Structural and functional findings in patients with moderate diabetic retinopathy

**DOI:** 10.1007/s00417-021-05277-y

**Published:** 2021-07-15

**Authors:** A. Boned-Murillo, M. D. Diaz-Barreda, A. Ferreras, I. Bartolomé-Sesé, E. Orduna-Hospital, P. Montes-Rodríguez, J. Ascaso, Isabel Pinilla

**Affiliations:** 1 Department of Ophthalmology, Lozano Blesa University Hospital, 50009 Zaragoza, Spain; 2 Aragón Health Research Institute (IIS Aragón), 50009, Zaragoza, Spain; 3 Department of Surgery, School of Medicine, University of Zaragoza, 50009 Zaragoza, Spain; 4 Department of Ophthalmology, Miguel Servet University Hospital, 50009 Zaragoza, Spain; 5 Department of Applied Physics, University of Zaragoza, 50009 Zaragoza, Spain

**Keywords:** Diabetic retinopathy, Microperimetry, Neurodegeneration, Swept-source OCT, Type 2 diabetes mellitus

## Abstract

**Purpose:**

To evaluate structural and functional ocular changes in patients with type 2 diabetes mellitus (DM2) and moderate diabetic retinopathy (DR) without apparent diabetic macular edema (DME) assessed by optical coherence tomography (OCT) and microperimetry.

**Methods:**

This was a single-center cross-sectional descriptive study for which 75 healthy controls and 48 DM2 patients with moderate DR were included after applying exclusion criteria (one eye per patient was included). All eyes underwent a complete ophthalmic examination (axial length, macular imaging with swept-source OCT, and MAIA microperimetry). Macular thicknesses, ganglion cell complex (GCC) thicknesses, and central retinal sensitivity were compared between groups, and the relationships between the OCT and microperimetry parameters were evaluated.

**Results:**

Macular thickness was similar in both groups (242.17 ± 35.0 in the DM2 group vs 260.64 ± 73.9 in the control group). There was a diminution in the parafoveal area thickness in the DM2 group in the GCC complex. Retinal sensitivity was reduced in all sectors in the DM2 group. The central global value was 24.01 ± 5.7 in the DM2 group and 27.31 ± 2.7 in the control group (p < 0.001). Macular integrity was 80.89 ± 26.4 vs 64.70 ± 28.3 (p < 0.001) and total mean threshold was 23.90 ± 4.9 vs 26.48 ± 2.6 (p < 0.001) in the DM2 and control group, respectively. Moderate correlations were detected between the central sector of MAIA microperimetry and retina total central thickness (− 0.347; p = 0.0035). Age, visual acuity, and hemoglobin A1c levels also correlated with retinal sensitivity.

**Conclusion:**

Macular GCC thickness and central retinal sensitivity were reduced in patients with moderate DR without DME, suggesting the presence of macular neurodegeneration. 
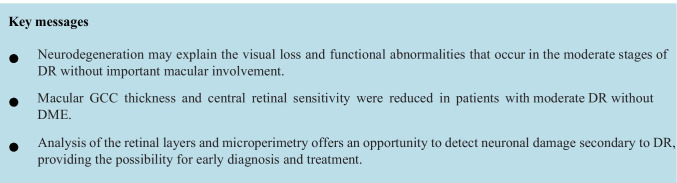

## Introduction

Diabetes mellitus (DM) is one of the most prevalent diseases of the twenty-first century and has great socio-sanitary importance, with an expected incidence of over 500 million in 2030 [1]. Diabetic retinopathy (DR) is a frequent and disabling complication, and is considered the most common cause of blindness in the active population of developed countries; diabetic macular edema (DME) is the most frequent cause of decreased visual acuity (VA) and proliferative DR is responsible for the most severe visual deficits [2, 3]. Good medical treatment for DM is therefore essential [4].

Regarding the pathogenesis of DR, microvascular changes are considered the most likely cause of visual loss, due mainly to chronic hyperglycemia [5]. A current topic of great interest, however, is neurodegeneration secondary to apoptosis of retinal cells as a potential cause, leading to progressive neuronal loss with atrophy and degeneration of the inner nuclear layer (INL), and the inner plexiform layer (IPL) and ganglion cell complex (GCC; comprising the retinal nerve fiber layer [RNFL], ganglion cell layer, and IPL), which would explain the changes observed prior to the microvascular findings [6–9]. In fact, some findings, such as a significant decrease in peripheral retinal sensitivity as well as RNFL thinning, suggest that neuronal damage is more related to diabetic neuropathy than to vascular changes [10]. These changes would explain the visual loss and functional abnormalities that occur in the early stages of the disease, prior to the onset of DR [11, 12].

In clinical practice, ophthalmologists rely mainly on the VA and macular thickness values obtained by optical coherence tomography (OCT) to establish the need to treat patients with DME, without considering other retinal function variables. Diabetic patients with normal VA and a normal-appearing fundus, however, may have changes at the functional level that alter macular sensitivity and functionality. Microperimetry to evaluate retinal sensitivity represents a selective method for evaluating early neuroretinal damage in this pathology [8]. Microperimetry improvements can track eye movement and to compensate them, allowing to assess retinal sensitivities in specific points and precise locations. It provides numerous data about the retinal sensitivity and can find subtle changes. Its sensibility and specificity make the microperimetry one of the most widely used and reliable method to check macular function.

The aim of our study was to compare the retinal sensitivity and structural changes measured by OCT between healthy individuals and patients with type 2 diabetes (DM2) having moderate DR without evidence of DME.

## Methods

The study protocol adhered to the tenets of the Declaration of Helsinki and approved by the Clinical Research Ethics Committee of the Hospital Clínico Universitario of Zaragoza and the Clinical Research Ethics Committee of Aragon (CEICA PI19/252). All participants provided written informed consent after receiving an explanation of the study.

### Study design

We conducted a descriptive, single-center cross-sectional study, including 141 eyes of 141 participants. Group 1 comprised 66 DM2 patients with moderate DR (graded by retinal specialists with color retinographs according to the ETDRS classification) and Group 2 comprised 75 healthy subjects with no previous ocular disease. Only one eye per subject was randomly selected. Patients were prospectively and consecutively recruited from the outpatient clinic of the Hospital Clínico Universitario from October 2018 through June 2019, more than 1 year after moderate DR diagnosis, and with good or moderate metabolic control (hemoglobin A1c (HbA1c) levels < 8%). The diagnosis of DR was established by fundus examination and retinography evaluation, according to the Global Diabetic Retinopathy Project Group criteria [13]. Inclusion criteria for the DM2 group were patients with moderate DR without actual or previous DME and the absence of another retinal pathology that could compromise the best-corrected VA (BCVA). Exclusion criteria were amblyopia or VA less than 20/40 on the Snellen chart, refractive error more than 5 diopters (D) of spherical equivalent (SE) or 3D of astigmatism, intraocular pressure (IOP) higher than 20 mmHg, history of any ocular pathology affecting central vision, patients with previous intraocular surgery, laser photocoagulation or anti-VEGF treatment, patients with topical glaucoma therapy as well as glaucoma with perimetric involvement or papillary atrophy, or inability to perform good quality OCT or microperimetry exams (difficulty in the layer segmentation, media opacification, or lack of fixation or cooperation—a total amount of 18 eyes were excluded based on this criteria, resulting in final studied patients’ number 48). The control group included healthy subjects older than 45 years of age recruited from among the hospital staff, family members, and other relatives of the research staff.

### Study protocol

All the exams were performed on the same day and in a standardized order. All participants underwent a complete ophthalmological evaluation, including BCVA expressed in logarithm of the minimum resolution angle (logMAR) measured with ETDRS at 4 m, IOP measured by Goldmann tonometry, and axial length (AL) using an Aladdin KR-1 W Series optical biometry system (Topcon Corporation, Tokyo, Japan) as the mean of 5 measurements and expressed in millimeters. In addition to the ophthalmological evaluation, a complete history was performed in which all aspects related to the patient’s disease (DM2) were evaluated, including current medication, time of evolution, HbA1c levels, lipid levels, glomerular filtration, and creatinine levels (the values were obtained within a period of less than 6 months of the examination).

Retinal thickness was evaluated using a swept-source OCT (SS-OCT), with the DRI-Triton SS-OCT (Topcon Corporation), and patient eyes were dilated prior to OCT imaging. For macular layer segmentation, we used the macular protocol (3D Macula H) and the IMAGEnet 6 Version software 1.22.1.14101® 2014 (Topcon Corporation), which performs measurements on a 100-cell grid located in the macula, each cell comprising 600 × 600 μm, collecting the macular thickness in each area of the center and 8 quadrants of the ETDRS grid. The regional retinal thicknesses, including the fovea (1 mm, C); the parafoveal ring with 4 quadrants, inner temporal (IT), inner superior (IS), inner nasal (IN), and inner inferior (II); and 4 perifoveal quadrants, outer temporal (OT), outer superior (OS), outer nasal (ON), and outer inferior area (OI).

**Fig. 1:**
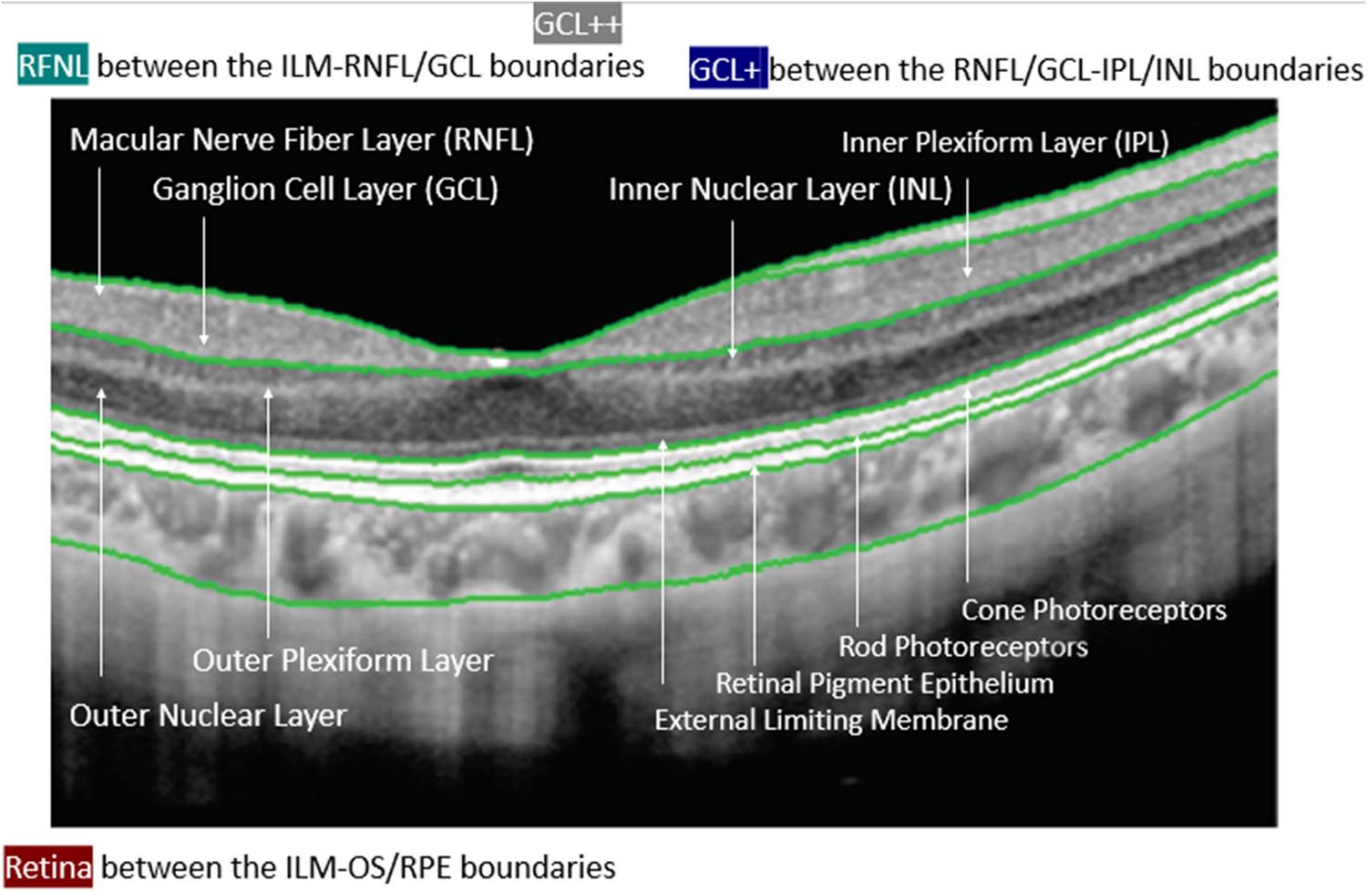
OCT for showing the total retina (from ILM to the boundary between the retinal pigment epithelium [RPE] and the photoreceptor layer [OS/RPE limit]), GCL + protocol (from the internal boundary of the ganglion cell layer [GCL; line RNFL/GCL] up to the external limit of the IPL [the IPL/INL line]), and GCL++ protocol (from the internal limiting membrane to the IPL/INL line [GCC]) used in the study

The DRI-Triton OCT performed automatic segmentation of the retinal layers, which was checked by a researcher performing a repositioning with “Grid, Reposition” function based on the reference retinal image if necessary. Three protocols were considered: total retina thickness (from ILM to the boundary between the retinal pigment epithelium [RPE] and the photoreceptor layer [OS/RPE limit]), GCL + protocol (from the internal boundary of the ganglion cell layer [GCL; line RNFL/GCL] up to the external limit of the IPL [the IPL/INL line]), and GCL +  + protocol (from the internal limiting membrane to the IPL/INL line [GCC]) (Fig. 1). Thicknesses of the described ETDRS areas of each retinal protocol were analyzed.

Finally, all patients were evaluated with third-generation microperimetry (Macular Integrity Assessment Device [MAIA]; Topcon Corporation). This device uses Goldmann III stimulation, projecting light stimuli directly onto the retinal surface with a size of 26 arc minutes and presented to the patient in different locations, attenuated in ranges from 0 to 36 dB. It also has a backlight of 1.27 cd/m^2^ (4 asb) and maximum luminance of 318.47 cd/m^2^ (1000 asb). A complete evaluation (expert) was carried out with a 4–2 complete threshold strategy, requiring a mean of 5.5 min, covering an area of 10° in diameter, including 37 measurement points, and thus obtaining an image composed of 3 radii of 12 points each and a foveal central point, all centered on the preferred retinal locus and with a radius of 1°, 3°, and 5°, respectively.

**Fig. 2:**
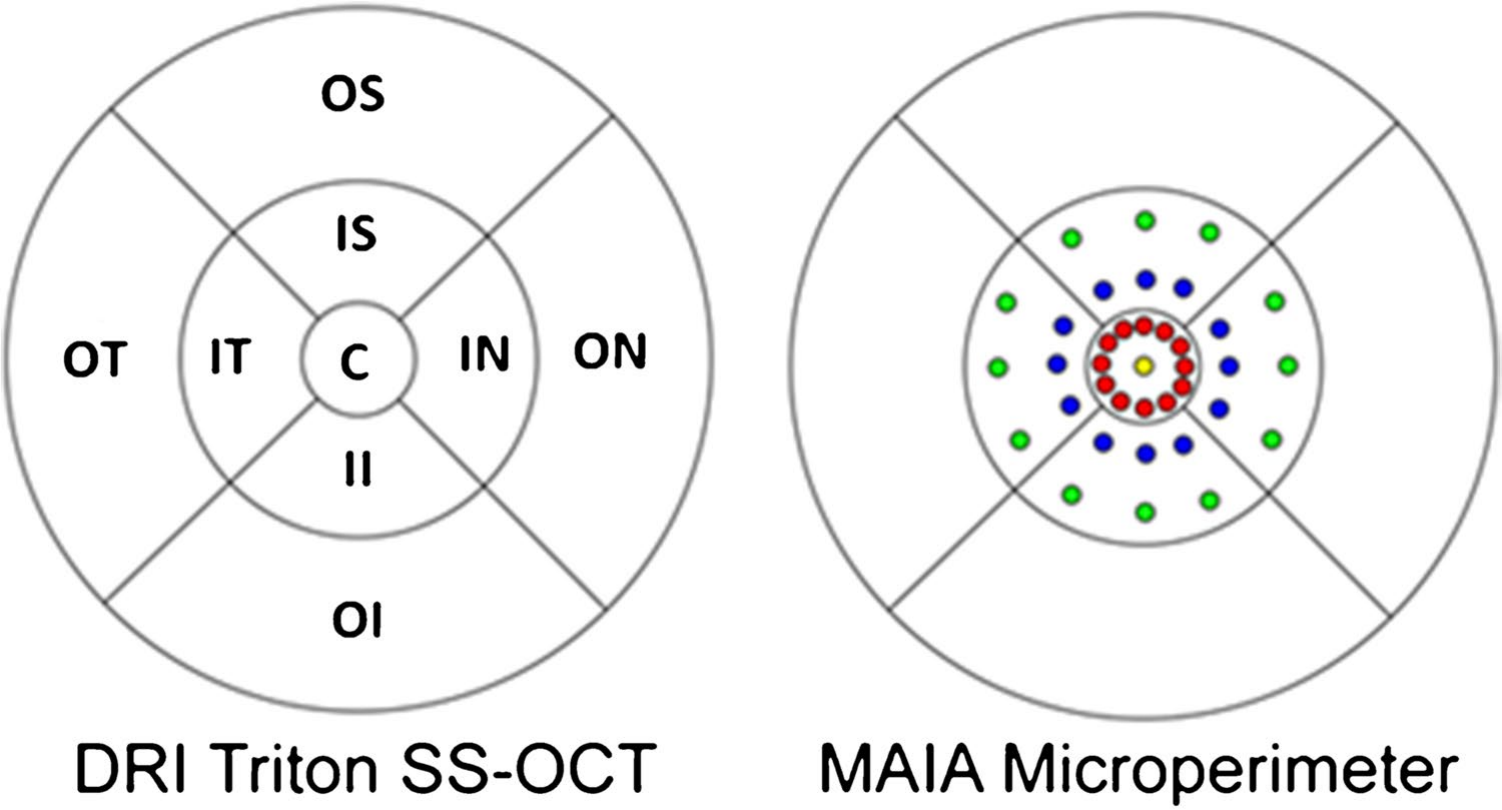
Grids of macular sectors for the DRI-Triton swept- source OCT (SS-OCT) and MAIA microperimeter for the 9 areas of the ETDRS grid (OS, outer superior; OT, outer temporal; OI, outer inferior; ON, outer nasal; IS, inner superior; IT, inner temporal; II, inner inferior, IN, inner nasal; C, central)

To evaluate the relationship of the sensitivity parameters provided by the MAIA with the OCT retinal thickness data, the MAIA sensitivity points were divided into sectors similar to those provided by the ETDRS grid. Considering an emmetropic eye, 1° in MAIA is approximately equivalent to a circle with a radius of 0.3 mm; 3° to a circle with a radius of 0.9 mm, and 5° to a circle with a radius of 1.5 mm. According to the measurements provided by the ETDRS grid, [14] the center point and the sensitivity points of 1° (0.6 mm diameters) were included in the central ring of the ETDRS, and the sensitivity points were located at 3° and 5° (diameters of 1.8 and 3 mm, respectively) to the 3 mm circle of the ETDRS grid. Thus, the mean of the retinal sensitivity thresholds calculated for the central MAIA and internal ring was arranged in the central ring of the ETDRS and the thresholds of the 3° and 5° radii corresponded to the upper, nasal, lower, and temporal internal quadrants of the ETDRS parafoveal ring (mean of 6 sensitivity points/quadrant; Fig. 2). The following results were obtained: fixation loss, retinal sensitivity, macular integrity index (classifies the age-related sensitivity deterioration when compared with the patient’s age adjusted normal values in normal if the loss is less than 40%, suspicious if the loss is between 40 and 60%, or as pathological (those due to AMD and other pathologies of the central retina) whose loss is greater than 60%), mean total threshold, fixation stability (p1 and p2, percentage of fixation points with respect to the total that are within a circle of 1° and 2° of radius, respectively, classified as stable [p1 > 75%], relatively unstable [p1 < 75% and p2 > 75%], or unstable [p2 is < 75%]), and bivariate contour ellipse area (BCEA)63 and BCEA95 (analysis of the area of an ellipse that contains all the fixation points, comprising 63% [BCEA63 or minor ellipse] and 95% [BCEA95 or greater ellipse] of the fixation points). The location of the fixation was checked manually and if the fixation was not properly centered on the fovea, the eye was excluded from the study. The fundoscopic image used to record the position of the stimuli was exported, extracting the perimetric information in a file that was subsequently imported to the SS-OCT for later analysis and comparison. Once the data were collected, the results were exported and transformed to be included in an Excel database (Microsoft Corporation, Redmond, WA, USA).

### Statistical analysis

For this study, all the variables were collected in spreadsheets in the Excel 2016 program and the information was subsequently imported into the Statistical Package for Social Sciences software (SPSS 20, SPSS Inc., IBM Corporation, Armonk, NY, USA) for statistical analysis. The results are expressed as mean and standard deviation for the quantitative variables, and as the number of cases and percentage for the qualitative variables. The Kolmogorov–Smirnov test was performed, verifying that the variables did not follow a normalized distribution. Thus, non-parametric tests were used for the analysis.

First, a descriptive analysis of the sample was carried out according to demographic variables and clinical characteristics. Second, to determine differences in the parameters between the control group and the DM2 group, the Mann–Whitney U test was used for independent samples. Correlations between anatomical and functional variables were also calculated by the Spearman correlation test. The Bonferroni correction for multiple comparisons was applied, resulting in a significance level of p < 0.004. A regression analysis was performed looking for the most relevant predictors of the global retinal sensitivity in DR patients.

## Results

### Clinical characteristics

Mean (SD) age was 62.7 (8.5) years (50 to 83 years) in the control group and 64.1 (10.7) years (42 to 84 years) in the DM2 group (p = 0.38). Table 1 shows the clinical characteristics of the sample. BCVA, SE, and AL were similar between groups; BCVA was slightly lower in the DM2 group without achieving statistical differences. IOP values were higher in the DM2 group.

**Table 1:** Clinical characteristics of the DM2 and control groups. Values are expressed as mean ± standard deviation (SD), and minimum–maximum values in brackets. The differences that reached statistical significance are shown in bold (p < 0.004). *DM*, diabetes mellitus; *BCVA*, best-corrected visual acuity; *SE*, spherical equivalent; *AL*, axial length; *IOP*, intraocular pressure; *HDL*, high-density lipoprotein; *LDL*, low-density lipoprotein; *TG*, triglycerides; *GFR (CKD-EPI)*, glomerular filtrate rate (Chronic Kidney Disease Epidemiology Collaboration)

	**DM2**	**Control**	
	Mean ± SD	Mean ± SD	p
BCVA (logMAR)	0.11 ± 0.16 (0.00 to 1.00)	0.06 ± 0.08 (0.00 to 0.30)	0.059
SE (D)	0.43 ± 2.13 (− 5.25 to 5.75)	0.65 ± 2.09 (− 9.25 to 4.62)	0.351
AL (mm)	23.35 ± 1.01 (22. 04 to 26.89)	23.58 ± 1.01 (22.01 to 27.74)	0.073
IOP (mmHg)	14.53 ± 2.63 (10 to 20)	13.15 ± 2.44 (8 to 19)	**0.002**
Years of DM evolution	3.30 ± 3.38 (0 to 11)		
HbA1c values (%)	7.80 ± 1.37 (5.30 to 7.7)		
Total cholesterol (mg/dL)	150.67 ± 30.65 (101.0 to 244.0)		
HDL cholesterol (mg/dL)	46.79 ± 13.65 (29.0 to 78.0)		
LDL cholesterol (mg/dL)	81.03 ± 55.77 (32.2 to –490.0)		
TG (mg/dL)	146.80 ± 99.14 (61.0 to 626.0)		
GFR (CKD-EPI) (mL/min/1,73)	70.70 ± 19.16 (28.53 to 90.0)		
Creatinine(mg/dL)	1.05 ± 0.39 (0.58 to 2.06)		

### Retinal thickness study (OCT): comparative analysis between the control and DM2 groups

**Fig. 3:**
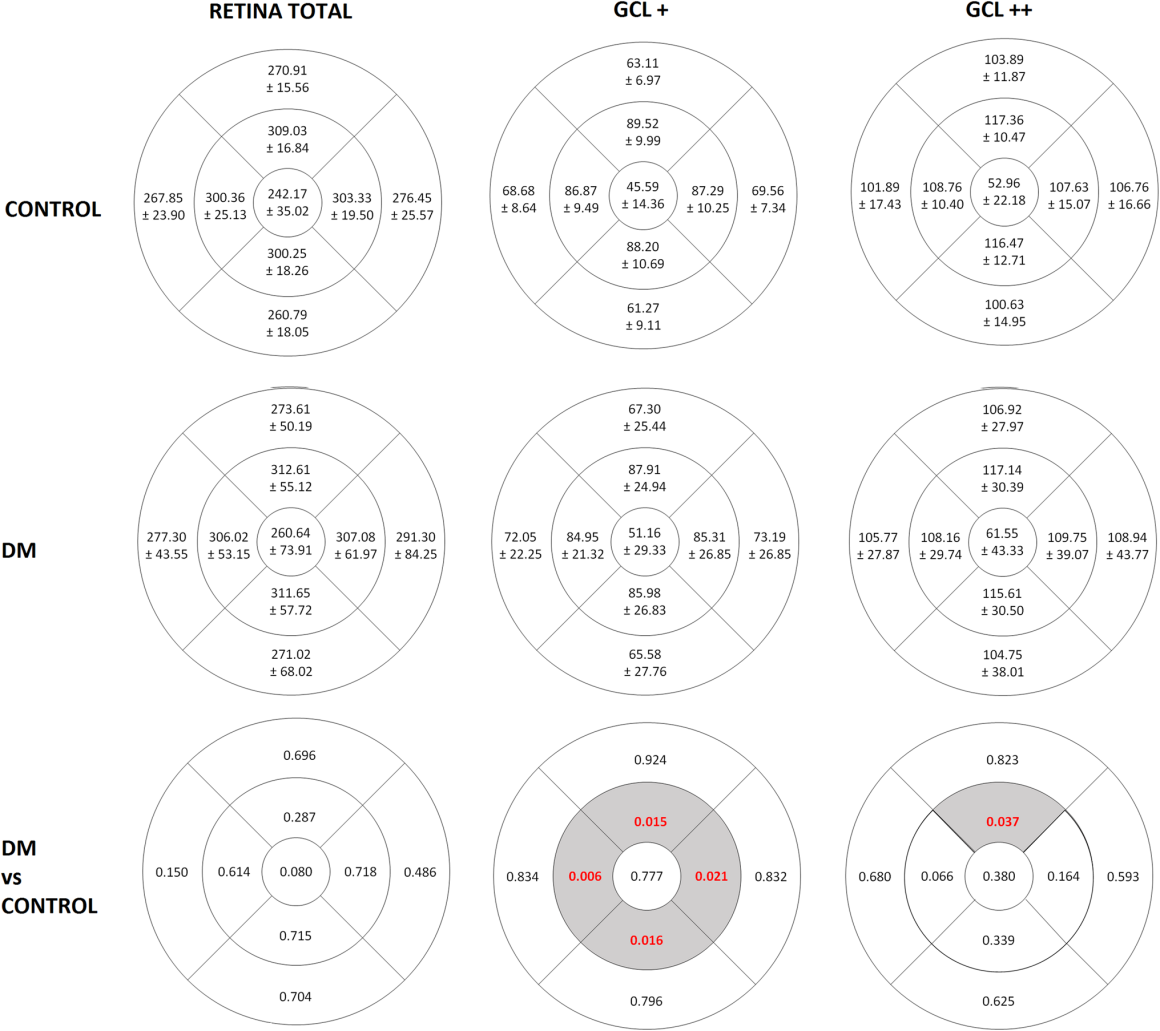
Mean and standard deviation (SD) of the thicknesses (in micrometers, µm) (first and second row) and p-values (last row) of the total retina layers, GCL + and GCL +  + protocols measured by the SS-OCT DRI-Triton in patients with DM2 and in healthy controls (significant values are highlighted in a different color). OS, outer superior; OT, outer temporal; OI, outer inferior; ON, outer nasal; IS, inner superior; IT, inner temporal; II, inner inferior; IN, inner nasal; C, central; RNFL, retinal nerve fiber layer

Macular thickness was similar in both groups (Fig. 3). Mean (SD) central macular thickness was 242.17 ± 35.0 in the DM2 group and 260.64 ± 73.9 in the control group. When assessed with the GCL + protocol, the thickness was reduced in the DM2 group compared with the control group in all the parafoveal areas, including the IS (p = 0.015), IT (p = 0.006), II (p = 0.016), and IN (p = 0.021) areas. With the GCL +  + analysis, only the IS area was decreased in the DM2 group compared with healthy individuals (p = 0.037).

### Retinal sensitivity using MAIA

**Fig. 4:**
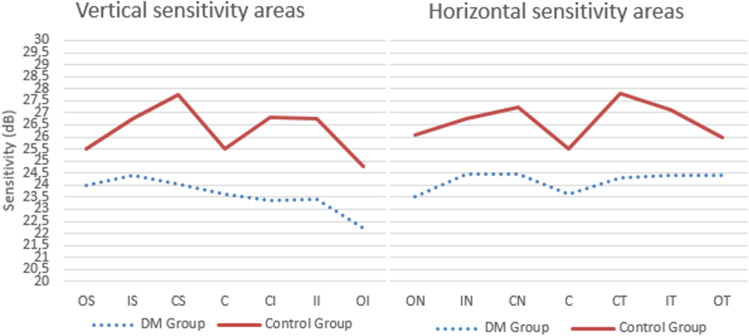
Mean retinal sensitivity in dB measured by the MAIA microperimeter for the 9 areas of the ETDRS grid (OS, outer superior; OT, outer temporal; OL, outer lower; ON, outer nasal; IS, inner superior; IT, inner temporal; II, inner inferior; IN, inner nasal; and C, central), which is then subdivided into CS: central superior, CT: central temporal, CI: central inferior, and CN: central nasal). All the values were significantly different between groups (p < 0.004)

MAIA microperimetry revealed lower retinal sensitivities in the DM2 group (Table 2, Fig. 4). The C global value (mean [SD] value of the central area and the inner ring) was 24.01 ± 5.7 in the DM2 group and 27.31 ± 2.7 in the control group (p < 0.001). The macular integrity (80.89 ± 26.4 vs 64.70 ± 28.3; p < 0.001) and total mean threshold (23.90 ± 4.9 vs 26.48 ± 2.6; p < 0.001) differed between the DM2 and the control groups, respectively (Table 3).

**Table 2:** Mean and standard deviation (SD) of the retinal sensitivity measured by MAIA microperimetry in diabetes mellitus 2 (DM2) patients and in healthy controls. Values are provided for the 9 areas of the ETDRS grid (*OS*, outer superior; *OT*, outer temporal; *OI*, outer inferior; *ON*, outer nasal; *IS*, inner superior; *IT*, inner temporal; *II*, inner inferior; *IN*, inner nasal; *C*, central), which is subdivided into *CS*, central superior; *CT*, central temporal; *CI*, central inferior; *CN*, central nasal; *C global*, central global). The values that reached statistically significant differences (p < 0.004) are shown in bold print

Retinal sensitivity (dB)			
	DM2 Media ± SD	Control Media ± SD	p
OS	23.98 ± 5.06	25.48 ± 3.04	**0.016**
OT	24.40 ± 6.27	25.97 ± 2.85	0.001
OI	22.20 ± 6.59	24.75 ± 3.64	0.002
ON	23.50 ± 5.82	26.09 ± 2.56	< 0.001
IS	24.39 ± 4.70	26.75 ± 2.55	< 0.001
IT	24.39 ± 5.60	27.10 ± 2.32	< 0.001
II	23.43 ± 6.88	26.77 ± 5.86	0.001
IN	24.44 ± 5.34	26.78 ± 2.71	0.001
C	23.62 ± 4.71	25.52 ± 3.37	**0.005**
CS	24.02 ± 6.03	27.73 ± 2.58	< 0.001
CT	24.28 ± 6.01	27.81 ± 2.64	< 0.001
CI	23.39 ± 6.58	26.79 ± 3.34	< 0.001
CN	24.48 ± 6.37	27.25 ± 3.00	**0.003**
C global	24.01 ± 5.74	27.31 ± 2.70	< 0.001

**Table 3:** Mean and standard deviation (SD) of the sensitivity of the variables measured by MAIA microperimetry in patients with diabetes mellitus (DM2) and in healthy controls and their comparison. *BCEA*, bivariate contour ellipse area. The values that reached statistical significance appear in bold print (p < 0.004)

Retinal sensitivity (dB)			
	DM2	Control	
	Media ± SD	Media ± SD	p
Macular integrity	80.89 ± 26.38	64.70 ± 28.31	** < 0.001**
Average threshold	23.90 ± 4.87	26.48 ± 2.55	** < 0.001**
Fixation stability P1	75.38 ± 28.52	83.27 ± 18.14	0.535
Fixation stability P2	86.74 ± 22.11	94.89 ± 7.27	0.118
BCEA 63 angle	6.97 ± 52.71	3.31 ± 61.16	0.939
BCEA 95 area	17.92 ± 32.13	10.02	0.270
BCEA 95 angle	3.57 ± 51.91	61.16 ± 45.22	0.790
Fixation loses (%)	8.98 ± 18.79	12.28 ± 20.31	0.529

### Regression analysis

The results (B coefficient (95%CI)) revealed that the most relevant predictors of global retinal sensitivity in DM patients were SE sensitivity (0.093 [0.032]; p = 0.005), IE (0.173 [0.037]; p < 0.001), CS (0.154 [0.032]; p < 0.001), CT (0.148 [0.031]; p < 0.001), and CI (0.135 [0.029]; p = 0.002).

### Correlation between the functional and anatomical values

Correlations for the clinical data and the functional and anatomical outcomes are shown in Table 4. Age, BCVA, and HbA1c had mild to moderate correlations with the OS area of retinal sensitivity measured by microperimetry. BCVA correlated moderately with all the retinal sensitivity parameters assessed by microperimetry.

**Table 4:** Correlations between retinal sensitivity measured by MAIA microperimetry and clinical variables in DM2 patients. *MP OS*, microperimetry outer superior; *OT*, microperimetry outer temporal; *OI*, microperimetry outer inferior; *ON*, microperimetry outer nasal; *IS*, microperimetry inner superior; *IT*, microperimetry inner temporal; *II*, microperimetry inner inferior; *IN*, microperimetry inner nasal; *MP C global*, microperimetry central global; *DR*, diabetic retinopathy; *HDL*, high-density lipoprotein; *LDL*, low-density lipoprotein; *TG*, triglycerides; *GFR (CKD-EPI)*, glomerular filtrate rate (Chronic Kidney Disease Epidemiology Collaboration); *BCVA*, best-corrected visual acuity; *SE*, spherical equivalent; *AL*, axial length; *IOP*, intraocular pressure. The values that reached statistical significance are shown in bold print (p < 0.004)

Retinal sensitivity																		
	MP OS		MP OT		MP OI		MP ON		MP IS		MP IT		MP II		MP IN		MP C global	
	dB	p	dB	p	dB	p	dB	p	**dB**	p	dB	p	dB	p	dB	p	dB	P
Age	− 0.323	0.009	** − 0.411**	0.001	** − 0.403**	0.001	− 0.321	0.009	** − 0.373**	0.002	− 0.242	0.054	− 0.298	0.017	− 0.282	0.024	− 0.266	0.033
Time evolution DR	0.021	0.869	0.064	0.610	0.097	0.442	− 0.146	0.246	− 0.048	0.709	0.023	0.859	− 0.041	0.747	− 0.072	0.574	0.009	0.942
HbA1c	− 0.205	0.101	− 0.157	0.211	− 0.208	0.097	** − 0.260**	0.036	− 0.228	0.070	− 0.132	0.299	** − 0.265**	0.035	− 0.229	0.069	− 0.212	0.093
Cholesterol	− 0.005	0.965	0.010	0.935	0.042	0.741	0.079	0.530	0.006	0.961	0.072	0.571	− 0.098	0.442	− 0.056	0.661	− 0.043	0.738
HDL	− 0.093	0.462	− 0.100	0.430	− 0.111	0.377	− 0.060	0.633	− 0.010	0.940	− 0.087	0.493	− 0.120	0.346	− 0.031	0.809	− 0.073	0.567
LDL	0.005	0.968	0.003	0.981	0.088	0.485	0.168	0.181	0.037	0.773	0.114	0.368	− 0.030	0.816	0.023	0.855	0.032	0.811
TG	0.046	0.715	0.081	0.521	0.006	0.964	− 0.027	0.830	− 0.046	0.719	0.029	0.822	− 0.063	0.621	− 0.092	0.470	− 0.069	0.585
GFR	− 0.040	0.753	0.058	0.645	− 0.044	0.729	0.057	0.652	− 0.049	0.699	0.018	0.890	− 0.067	0.597	− 0.017	0.894	− 0.078	0.538
Creatinine	0.214	0.087	0.036	0.777	0.190	0.129	0.145	0.248	0.201	0.111	0.118	0.355	0.187	0.138	0.089	0.487	0.163	0.198
BCVA	** − 0.594**	< 0.001	** − 0.460**	< 0.001	** − 0.430**	< 0.001	** − 0.421**	< 0.001	** − 0.561**	< 0.001	** − 0.508**	0.000	** − 0.516**	< 0.001	** − 0.580**	0.000	** − 0.539**	< 0.001
S.E	− 0.258	0.038	− 0.203	0.105	− 0.384	0.020	− 0.323	0.009	− 0.318	0.010	− 0.183	0.149	− 0.322	0.010	− 0.234	0.063	− 0.315	0.110
A.L	0.138	0.273	0.100	0.428	0.222	0.076	0.251	0.044	0.220	0.081	0.149	0.241	0.242	0.054	0.190	0.133	0.281	0.025
IOP	− 0.036	0.776	− 0.034	0.789	− 0.079	0.533	− 0.141	0.264	− 0.003	0.982	− 0.092	0.468	− 0.094	0.460	− 0.029	0.821	− 0.099	0.436

Central macular thickness (OCT) and retinal sensitivity of the inner ring (MAIA) were moderately correlated (r =  − 0.347; p = 0.0035) (Table 5). No significant correlations were observed between retinal sensitivity of internal and external quadrants measured by MAIA microperimetry and retinal thickness of the internal ring measured by SS-OCT in patients with type 2 diabetes.

**Table 5:** Correlations between retinal sensitivity of central quadrants measured by MAIA microperimetry and retinal thickness of the central ring measured by SS-OCT in patients with type 2 diabetes. *GCL* + , from the internal boundary of the ganglion cell layer [GCL; line RNFL/GCL] up to the external limit of the IPL [the IPL/INL line]); *GCL* +  + , from the internal limiting membrane to the IPL/INL line [GCC]); *C*, central; *C global*, central global. Bold numbers indicate statistically significant differences (p < 0.004)

Retinal sensitivity	ETDRS C (total retina)	ETDRS C (GCL +)	ETDRS C (GCL + +)
C global	− 0.347	− 0.246	− 0.283
p	**0.0035**	0.054	0.026

## Discussion

Our results suggested functional deterioration at the macular level in patients with DR without DME. Although some investigators have argued that retinal neurodegeneration is only a manifestation of diabetic polyneuropathy, other researchers [9] consider that signs of retinal neurodegeneration are observed prior to microvascular alterations, as described by Simó et al. [15]. Our group has already described anatomical and functional alteration prior to the appearance of DR. In the present study, we were looking for these neurodegeneration signs in patients with already developed DR but with not comprised macular function by DME. The link between the processes, however, remains unknown. In addition to vascular alterations, multiple early changes in the neuroretina of DM patients have been observed [19]. These structural changes have been preferentially demonstrated in patients with evidence of microvascular lesions and reduction of the GCL and RNFL thickness, [16–18] as well as in other studies of DM patients without signs of DR or with minimal alterations but with structural findings suggesting neurodegeneration. In our study, the mean (SD) BCVA in the DM2 group with moderate DR and no DME was good (0.11 [0.2] logMAR), with better values for the control group but no significant difference between them (p = 0.06), indicating well-preserved visual function in terms of VA.

Regarding macular thickness, we detected no differences between groups in any of the quadrants of the ETDRS. The fovea (ETDRS C) had the lowest thickness in both groups (242.17 [35.02] μm in the DM2 group and 260.64 [73.91] μm in the control group) and the thinnest sector was the outer inferior (260.79 [18.05] μm in the DM2 group and 271.02 [68.06] μm in the control group). As reported, the inner macular areas had greater thicknesses than the outer macular areas, the nasal greater than the temporal, and the superior greater than the inferior [19]. Analysis of retinal thickness in patients with DR compared to healthy patients reveals wide variability in the results due to changes related to the disease evolution and the absence of a uniform pattern or behavior [20–23]. The thickness decreases due to the loss of neural tissue, although it could increase due to vascular permeability and inflammation, thus counteracting the effect of neurodegeneration on macular thickness, as Sugimoto et al. [24] suggested. But increased macular thickness is not sufficient to rule out the existence of an associated neurodegenerative process [25]. Macular thickness cannot be considered the only parameter for evaluating early changes in the retina of DM2 patients, because is not sensitive enough to detect changes in the microstructure of the retinal layers.

In our study, the inner sectors of the GCL + (GCL + IPL complex) showed thinning in the DM2 group with differences between groups (IS, p = 0.015; IT, p = 0.006; II, p = 0.016; and IN, p = 0.021), which is consistent with the results published by Bandello et al. [26]. Other investigators, such as Ng et al., [27] Scarinci et al., [28] and Gundogan et al. [29] considered that measurement of the GCL is preferable to that of the RNFL for quantifying the loss of ganglion cells, because the ganglion cell bodies have a diameter 10–20 times larger than that of their axons that form the RNFL and have different metabolic needs, which could produce different results despite the same damage [26, 27]. In the Chhablani et al. [30] study, the mean RNFL thickness in the macula did not differ significantly between a control group and 3 types of diabetic patients (with no DR, with non-proliferative DR, and with proliferative DR), whereas the GCL-IPL complex was significantly thinner in all patients with diabetes, including those without DR. Additionally, they found a greater thinning of the nasal area of the GCL + , where the bundle of fibers goes to the optic nerve head, which is similar to what occurs in neurodegenerative diseases.

On the other hand, when we evaluated the thickness of the layers included in the GCL +  + protocol (GCC), the inner superior area showed significant thinning in the DM2 group (p = 0.037). In this regard, another study carried out in patients with DM2 without DR detected a reduction of the GCC in the upper macular area. They found that the thickness of the macular GCC was decreased in DM patients before loss of the peripapillary RNFL. Other studies have examined changes in the retinal layers. Tavares Ferreira et al. analyzed the outermost layers of the retina in the macular area and found a significant reduction in the photoreceptor layer of DM patients compared to healthy subjects [31]. Salvi et al. [9] analyzed the GCC in patients with DM2 and healthy individuals and found no differences between the groups.

We correlated microperimetry points with the corresponding areas in the ETDRS grid. We did not consider the displacement of the GC from their receptive fields because there is no clear agreement due to the variability between subjects [32, 33]. In our study, the DM2 group had reduced retinal sensitivity compared with the control group, similar to previous reports [4, 7, 8, 34]. Nittala et al. [8] reported lower retinal sensitivity in patients with severe DR than in those with mild or moderate DR. In addition, they observed that the sensitivity of the central 20° of the macula was reduced depending on the severity of the DR, being lower in patients with severe non-proliferative DR than in patients with proliferative DR. They also found that in patients with DM without DR, there is a loss of retinal sensitivity between the center, 2°, and 20° (2°, 8°, 10°, 12°, 20°), as well as in the lower and nasal quadrants, but not in the temporal quadrants.

We observed a negative correlation of age and BCVA with retinal sensitivity. Other studies, such as those of Okada et al. [35] and Nittala et al., [8] reported that reduced retinal sensitivity was related to an increase in the foveal avascular zone and in the duration and severity of the disease, as well as morphological alterations detected with OCT. Other authors, however, reported no significant relationship [36]. The loss of retinal sensitivity with age has been also described in healthy people, with a mean reduction of 0.11 dB/year using the MP-1 microperimeter [8]. We also observed a negative correlation between the HbA1c levels and retinal sensitivity in the ON and II areas, which suggests that worse control of the disease is related to a worse prognosis and increases the risk for developing DME, as reported by van Dijk et al., Martin et al., and Verma et al. [7].

The central inner ring of the MAIA perimetry correlated with the central macular thickness (r =  − 0.347; p = 0.005). The results are similar to other studies, like Verma et al., [7] Senthil et al. [37], and Orduna-Hospital et al. [38], which also found a correlation between the foveal thickness and retinal sensitivity, while other authors also evaluated the relationship between retinal sensitivity and the thickness of the retinal inner layers (GCL and IPL) between healthy and patients with DM2 without DR, and did not find such a correlation [4, 39].

The main limitations of our study were the size of the sample and how it only included patients with moderate DR. Future studies including a higher number of patients as well as different RD stages would further clarify the functional impairment of diabetic patients.

In conclusion, functional and anatomical alterations are observed in patients with DM with moderate DR, suggesting that DM leads to a deterioration in retinal sensitivity and inner retinal layer thickness, even though the vascular lesions are moderate and with no macular edema. Retinal thicknesses are diminished in DM2 patient’s inner retina, with no clear affection of the RNFL. Analysis of the retinal layers and microperimetry offer an opportunity for early detection of neuronal damage secondary to this pathology, providing the possibility for early diagnosis and improved prognosis.
